# Announcement

**DOI:** 10.1155/S1463924600000286

**Published:** 2000


					who on average have been with Schroder Ventures 9
years.
Schroder Ventures' funds have been investing in Eur-
opean healthcare businesses for 15 years. Notable prior
investments include Chiroscience, Shire Pharmaceuticals
and Micromass.
Schroder Ventures does not intend to accept any new
applications for commitments in the fund.
For futher information, contact: Schroder Ventures, Charles
Sherwood, Partner, London. Tel: + 44 ( 0) 20 7632 1032; or
Amanda Shaw, Marketing Manager, Tel: + 44 ( 0) 20 7632
1007/44 ( 0) 7979 706768.
Announcement
New CAMAG catalogue: Planar Chromatography
2000 - modern thin-layer chromatography
Planar Chromatography is a modern separation tech-
nique which is accepted by regulatory agencies and
analysts as an extremely  exible, reliable and cost-
e ective method. It is successfully used in pharmaceutical
industry, food analysis, natural products/herbals and
many other (R) elds of application.
Besides information on CAMAG products and services
the catalogue includes a brief guide to the operating
procedure of Planar Chromatography.
The catalogue contents and ancillary information on
CAMAG services can also be accessed in the Internet
under www.camag.ch
For further information please contact: Delphine Stoa el, Sales &
Marketing Administration, CAMAG, CH-4132 Muttenz/
Switzerland. Tel: + 41 61-467 34 34; Fax: + 41 61-461 07
02; e-mail: info@camag.ch
News/
Announcement
168

				

